# Effect of Erufosine on Membrane Lipid Order in Breast Cancer Cell Models

**DOI:** 10.3390/biom10050802

**Published:** 2020-05-22

**Authors:** Rumiana Tzoneva, Tihomira Stoyanova, Annett Petrich, Desislava Popova, Veselina Uzunova, Albena Momchilova, Salvatore Chiantia

**Affiliations:** 1 Bulgarian Academy of Sciences, Institute of Biophysics and Biomedical Engineering, 1113 Sofia, Bulgaria; tzoneva@bio21.bas.bg (R.T.); tihomira_stoyanova@abv.bg (T.S.); desislava.popova94@abv.bg (D.P.); vesi.uzunova@abv.bg (V.U.); albena_momchilova@abv.bg (A.M.); 2 Institute of Biochemistry and Biology, University of Potsdam, Karl-Liebknecht-Street 24-25, 14476 Potsdam, Germany; apetrich@gmx.net

**Keywords:** alkylphospholipids, fluorescence microscopy, fluorescence correlation spectroscopy, lipids, plasma membrane, cancer, lipid–lipid interactions, membrane microdomains, membrane biophysics

## Abstract

Alkylphospholipids are a novel class of antineoplastic drugs showing remarkable therapeutic potential. Among them, erufosine (EPC3) is a promising drug for the treatment of several types of tumors. While EPC3 is supposed to exert its function by interacting with lipid membranes, the exact molecular mechanisms involved are not known yet. In this work, we applied a combination of several fluorescence microscopy and analytical chemistry approaches (i.e., scanning fluorescence correlation spectroscopy, line-scan fluorescence correlation spectroscopy, generalized polarization imaging, as well as thin layer and gas chromatography) to quantify the effect of EPC3 in biophysical models of the plasma membrane, as well as in cancer cell lines. Our results indicate that EPC3 affects lipid–lipid interactions in cellular membranes by decreasing lipid packing and increasing membrane disorder and fluidity. As a consequence of these alterations in the lateral organization of lipid bilayers, the diffusive dynamics of membrane proteins are also significantly increased. Taken together, these findings suggest that the mechanism of action of EPC3 could be linked to its effects on fundamental biophysical properties of lipid membranes, as well as on lipid metabolism in cancer cells.

## 1. Introduction

Erufosine (EPC3) is a novel derivate of erucylphosphocholine that belongs to a group of antineoplastic drugs based on alkyl ether lipids [1]. EPC3 can effectively be applied intravenously, can cross the blood-brain barrier, and shows antitumor activity in the μM range [1,2]. For these reasons, EPC3 is a promising drug for treatment of several types of tumors, including human urinary bladder carcinoma, breast carcinoma, glioblastoma, and multiple myeloma [2]. On the one hand, due to its hydrophobic nature, this molecule is supposed to interact with cellular membranes, but detailed information regarding its molecular mechanism of action is scarce. On the other hand, other alkylphospholipids (APL) have been characterized in more detail. For example, it was shown that edelfosine, which was one of the first characterized APLs, induced apoptosis in cancer cells via interactions with lipid rafts [1], i.e., lipid-protein domains of the plasma membrane (PM) which are enriched in sphingolipids and cholesterol [3,4,5] and are involved in several cellular functions (see e.g., [6,7]). Such domains can also be characterized in protein-free model membrane systems (e.g., lipid vesicles) constituted of typical PM lipids (e.g., saturated sphingomyelin (SM), unsaturated phosphatidylcholine (PC), and cholesterol). It was shown in fact that these model membranes displayed a phase separation into a liquid-ordered (L_o_) and a liquid-disordered (L_d_) phase [8]. The L_o_ phase was enriched in saturated lipids and cholesterol, and therefore provided a simple physical model to study raft-like domains [9,10]. In this context, it was shown that APLs partitioned into lipid bilayers and directly interacted with L_o_ domains [11]. The observed effects varied depending on lipid composition and the investigated APL (e.g., edelfosine or miltefosine) and included: disorganization of L_o_ domains [11], moderate to significant increase of membrane fluidity [12,13], and stabilization of SM/cholesterol domains [14]. More specifically, studies on erucylphosphocholine (which is more similar to EPC3, due to the shared unsaturated acyl chain structure) have indicated that this APL increases the fluidity of both cellular and model membranes [12], while weakening SM–cholesterol interactions [15].

So far, studies regarding the molecular mechanisms connected to the antitumor activity of EPC3 have been limited. Recently, using spectroscopic ellipsometry as a novel technique to study solid-supported lipid model systems, we have shown that treatment of lipid films composed of PC, SM, and cholesterol with EPC3 induced an increase in monolayer thickness [16].

In this work, we employed several methods based on fluorescence microscopy and analytical chemistry to investigate, for the first time, how EPC3 affected the physical properties of the PM.

First, we applied line-scan fluorescence correlation spectroscopy (lsFCS) [17,18] and scanning fluorescence correlation spectroscopy (sFCS) [19,20,21] to monitor the dynamics of membrane lipids and proteins, both in simple artificial membranes and directly in the PM of living cells. These methods belong to the family of fluorescence fluctuation techniques and have been effectively used in the past to quantify membrane dynamics (and, indirectly, membrane order) [22]. Furthermore, supported lipid bilayers (SLBs) as membrane models were used to study lipid–lipid interactions in a controlled environment and in specific relation to the precise lipid composition of the bilayer [23,24].

Second, using thin layer chromatography and gas chromatography, we evaluated the changes occurring in phospholipid and cholesterol amounts in the membranes of EPC3-treated cancer cells.

Finally, we quantified the influence of EPC3 on membrane order for the PM of cancer cell models using polarity-sensitive fluorescent probes [25]. In more detail, we investigated the spectral properties of two membrane probes (Laurdan and Di-4-ANEPPDHQ) which were shown to be influenced by lipid packing, membrane hydration, and composition to different extents [25,26].

Our results indicate that EPC3 modulates the PM lipid composition of cancer cells. Furthermore, it affects lipid–lipid interactions both in lipid membrane models and cellular membranes. Such alterations in membrane order appear to have a direct effect on membrane protein dynamics.

## 2. Materials and Methods

### 2.1. Chemicals

Erufosine (EPC3) was synthesized in the Max Planck Institute for Biophysical Chemistry, Göttingen, Germany, and was most graciously provided by Prof. Martin R. Berger. It was dissolved in a PBS (phosphate buffer saline, 137 mM NaCl, 2.7 mM KCl, 8 mM Na_2_HPO_4_, and 2 mM KH_2_PO_4_, pH 7.4) in 10 mM stock solution and kept at 4 °C. Di-4-ANEPPDHQ and Laurdan (6-dodecanyl-2-dimethylaminonaphtalene) were from Molecular Probes (Eugene, OR, USA). l-α-phosphatidylcholine from chicken egg (eggPC), cholesterol from ovine wool (Chol), sphingomyelin from porcine brain (bSM), 23-(dipyrrometheneboron difluoride)-24-norcholesterol (TF-Chol) and 1,2-dioleoyl-sn-glycero-3-phosphoethanolamine-n-(lissamine rhodamine B sulfonyl) (Rhod–DOPE) were purchased from Avanti Polar Lipids, Inc. (Alabaster, AL, USA).

### 2.2. Cell Culture

MDA-MB-231 highly invasive breast cancer cells and MCF-7 epithelial cancer cells were acquired from the American Type Culture Collection (ATCC, Kielpin Lomianki, Poland). Both cell lines are derived from breast adenocarcinoma, with MCF-7 cells retaining some characteristics of the differentiated mammary epithelium. All cell lines were incubated in phenol red-free DMEM culture medium with 10% fetal bovine serum, 2 mM L-glutamine, and 100 U/mL penicillin and 100 μg/mL streptomycin at 37 °C and 5% CO_2_. Cells were passaged every 3–5 days, no more than 15 times. All solutions, buffers, and media used for cell culture were purchased from PAN-Biotech (Aidenbach, Germany).

### 2.3. Plasmids

The plasmid coding for human EGFR tagged with EGFP (hEGFR-EGFP) was a kind gift from Alexander Sorkin (Addgene plasmid #32751) [27]. To replace EGFP with mEGFP, both plasmids hEGFR-EGFP and mEGFP-N1 (gift from Michael Davidson, Addgene plasmid #54767) were digested with AgeI and NotI (New England Biolabs GmbH, Ipswich, MA, USA). The hemagglutinin (HA) gene from influenza virus strain A/FPV/Rostock/34 (H7N1) tagged with mEGFP at the C-terminus (FPV-HA-mEGFP) was cloned based on the previously described FPV-HA-mEYFP (gift from Andreas Herrmann, Humboldt University Berlin) [28]. Briefly, FPV-HA-mEYFP was digested using BglII and SacII (New England Biolabs GmbH, Ipswich, MA, USA), and the obtained HA-insert was ligated into mEGFP-N1. This plasmid is available on Addgene (#127810).

### 2.4. Supported Lipid Bilayers

Supported lipid bilayers (SLBs) were prepared as previously described [29,30]. Briefly, lipids were mixed in chloroform at the desired concentrations and dried on the walls of a glass vial. Then, the lipid film was rehydrated in PBS pH 7.4 and, after vigorous vortexing, sonicated to clarity in a bath sonicator. Typical concentrations during sonication were ~5–10 μM. Then, the obtained small unilamellar vesicle suspension was diluted ca. 10-fold and 100 μL were deposited on a small thin piece (~10 mm^2^) of freshly cleaved mica glued to the surface of a glass coverslip (thickness #1). The mica and the vesicles suspension were confined using a 7 mm plastic cylinder, also glued to the glass surface. Vesicle fusion and bilayer formation were induced by addition of 3 mM CaCl_2_. The volume was adjusted to 300 μL and the suspension was incubated for 10 min. Unfused vesicles were removed by addition and removal of 500 μL PBS, performed 10 times. The treatment with EPC3 was performed by adding the drug at the desired concentration in PBS directly on top of the SLB and waiting ca. 30 min before performing fluorescence measurements.

### 2.5. Line-Scan Fluorescence Correlation Spectroscopy on SLBs

The line-scan FCS (lsFCS) measurements on SLBs were performed as previously described [17]. Briefly, data were acquired by repeatedly scanning the focal volume in a linear fashion in the plane of the membrane. Line scans of ca. 5–10 μm length were chosen so that both bilayer phases (L_d_ and L_o_) were scanned through. We typically acquired 2.5 × 10^5^ lines, each divided in 256 pixels, with a pixel time of 1.27 μs (line time 763 ms). The intensity values were correlated along each line and between different lines, calculating the full spatiotemporal autocorrelation G(ξ, τ_i_). To account for photobleaching, a mathematical correction was applied [17]. Data analysis was performed with a custom-written script in Matlab, by fitting G(ξ, τ_i_) using a weighted nonlinear least-squares fitting algorithm and a mathematical model taking into account the linear scanning and two-dimensional (2D) Brownian diffusion. To capture the statistical information at larger lag times, we also included in the evaluation (via global fitting) the analysis of the temporal autocorrelation curve G(0, τ_i_), calculated on a logarithmic scale with a multiple τ-algorithm [17]. Thus, we could obtain estimates for the waist $\omega_{0}$ and the diffusion coefficient D for a fluorescent lipid analogue (TF-Chol, 0.01 mol%) added to the examined SLBs. The autocorrelation curves were calculated independently for the different lipid phases, which could be identified due to the distinct affinity of a fluorescent dye (i.e., Rhod-DOPE, 0.1 mol%) to the L_o_- and the L_d_-phases. Line scans were performed using the same setup described above for sFCS, using a 488 nm argon laser (ca. 1.5 μW) for the excitation of TF-Chol. The signal originating from the Rhod-DOPE (excitation 561 nm, 561/488 dichroic mirror, emission collected between 571 and 650 nm) was collected only in order to distinguish the lipid phases, but not used further for lsFCS analysis. The reported D values for TF-Chol were calculated as an average of ca. 10 measurements for each EPC3 concentration, from three independent experiments, performed in different days. The D values in the L_o_ and L_d_ phase of SLB in the absence of EPC3 were used as a normalization reference for the D values measured in the presence of EPC3, in order to emphasize the relative effect of the drug rather than, for example, day-to-day variations in sample properties.

### 2.6. Lipid Extraction and Analysis of Phospholipids

MCF-7 and MDA-MB-231 cells were seeded in 25 cm^2^ cell culture flasks at a density of 1.5 × 10^5^ cells/mL. After a 24 h incubation, cells were treated with IC_50_ and IC_75_ amounts of EPC3 [31] and further incubated for 24 h. The extraction of membrane lipids (including internal membranes) was performed as described previously [32] with chloroform/methanol, according to the method of Bligh and Dyer [33]. Briefly, the organic phase obtained after extraction was concentrated and analyzed by thin layer chromatography. The individual phospholipid fractions were separated on silica gel G 60 plates (20 × 20 cm, Merck, Darmstadt, Germany) in a solvent system containing chloroform/methanol/acetic acid/water (70:35:8:4, *v*/*v*). The location of the separated fractions was visualized by iodine staining. The spots were scraped and quantified by estimation of inorganic phosphorus [34].

### 2.7. Determination of Cholesterol by Gas Chromatography

The cholesterol content in the membranes of EPC3-treated breast cancer cells (as described above) was determined using gas chromatography [35] using Carlo Erba gas-chromatography equipped with a flame-ionization detector isothermally at 190 °C and with a 2 m column coated with 10% DEGS on Chromosorb W60/80 mesh (Pharmacia Fine Chemicals Inc., Piscataway, NJ, USA), with nitrogen as the gas carrier.

### 2.8. Confocal Microscopy Imaging Using Di-4-ANEPPDQ and Laurdan

The cells were seeded in 35 mm microscopy dishes (CellVis, Mountain View, CA, USA) with an optical glass bottom (#1.5 glass, 0.16–0.19 mm) at a density of 5 × 10^4^ cells/well for MDA-MB 231 and 1 x 10^5^ cells/well for MCF-7. After an ~12 h incubation, the MDA-MB-231 cells were treated with a 20 (or 30) μM EPC3 solution in culture medium, corresponding to the previously measured IC_50_ and IC_75_ values [31]. The MCF-7 cells were treated with a 40 (or 60) μM EPC3 solution (corresponding to IC_50_ and IC_75_ values, Tzoneva R., unpublished data) in culture medium. In both cases, the treatment lasted for 24, 48, or 72 h. Control cells were incubated just with culture medium, following the same protocols. After the incubation period, the cell medium was removed. Then, the cells were washed twice with PBS, pH 7.4. Afterwards, 2 mL of serum-free and phenol red-free DMEM, and fluorescent dye (with final concentration of 1 μM for Di-4-ANEPPHQ or 5 μM for Laurdan) were added to the cell dish. The cells were further incubated for 30 min at 37 °C.

A Zeiss LSM 780 system (Carl Zeiss, Oberkochen, Germany) was used to acquire the confocal images, with a pixel size of ca. 200 nm (512 × 512 pixels). Samples were imaged using a Plan-Apochromat 40×/1.2 Korr DIC M27 water immersion objective. The excitation sources were a 488 nm argon laser (for Di-4-ANEPPDQ) or a 405 nm diode laser (for Laurdan). Fluorescence was detected between 498–579 nm (Channel 1) and 620–750 nm (Channel 2) for Di-4-ANEPPDQ, after passing through a 488 nm dichroic mirror, using a gallium arsenide phosphide (GaAsP) detector. Fluorescence of Laurdan was detected in the spectral ranges 410–463 nm (Channel 1) and 472–543 nm (Channel 2), after passing through a 405/565 nm dichroic mirror. Out-of-plane fluorescence was reduced by using a 42.4 μm pinhole in front of the detector.

Confocal images were analyzed as previously described [25,26]. Briefly, for each experimental condition, ca. 5–10 confocal images of treated cells were acquired. In each fluorescence intensity image, several regions of interest (ROIs) were selected in correspondence of the PM of different cells. Due to the effect to the EPC3 treatment, some cells displayed significant morphological alterations, while undergoing apoptosis. We focused our analysis instead on pre-apoptotic cells, i.e., morphologically comparable to untreated cells. In these cells, ROIs could be chosen so to include portions of the PM clearly recognizable as ~0.5 μm thick bright lines (see Figure S1 for examples). Experiments were performed as independent duplicates on different days so that, for each EPC3 concentration and each time point, at least 50 cells were analyzed in total. All the pixels from ROIs collected within equivalent samples (i.e., same EPC3 concentration and same time point) were pooled together. For each pixel, the generalized polarization (GP) value was calculated as defined in [26], using a custom-written Matlab (The MathWorks, Natick, MA, USA) script and setting the calibration factor G = 1. The obtained results are shown as normalized occurrence histograms of all selected pixels.

### 2.9. Scanning Fluorescence Correlation Spectroscopy (sFCS) in Living Cells

For one-color sFCS experiments, 8 × 10^4^ MDA-MB-231 cells were seeded in 35 mm dishes (CellVis, Mountain View, CA, USA) with optical glass bottoms (#1.5 thickness, 0.16–0.19 mm). After 24 h, cells were treated with medium containing 30 μM EPC3. After an additional incubation for 24 h, cells were transfected by using 200 ng (mp-mEGFP and mp-mEGFP(2×)) or 600 ng (hEGFR-EGFP or A/FPV-HA-mEGFP) plasmid DNA per dish with Lipofectamine^TM^ 3000 according to the manufacturer’s instructions (Thermo Fisher Scientific, Waltham, MA, USA). Plasmids were incubated for 15 min with 2 μL P3000 per μg plasmid and 2 μL Lipofectamine^TM^ 3000 diluted in 50 μL serum-free medium, and then added dropwise to the cells.

After 24 h incubation, scanning fluorescence correlation spectroscopy (sFCS) measurements were carried out on a Zeiss LSM 780 system, equipped with a Plan-Apochromat 40×/1.2 Korr DIC M27 water immersion objective. Samples were excited with a 488 nm argon laser and the fluorescence was detected between 499 and 597 nm, after passing through a 488 nm dichroic mirror, using a GaAsP detector. To decrease out-of-focus light, a pinhole size of one airy unit (~39 μm) was used. To keep photobleaching below 20%, a laser power of 1.2 μW was chosen. For cell measurements, a line-scan of 256 × 1 pixel (pixel size 80 nm) was performed perpendicular to (i.e., across) the PM with a 403.20 μs scan time (0.67 μs pixel dwell time). For each measurement, 4 × 10^5^ lines were acquired in photon counting mode, and the total scan time was around 3 min per measurement. Scanning data were exported as TIFF files. At the beginning of each measurement day, the signal was optimized by adjusting the collar ring of the objective to the maximal count rate for an Alexa Fluor^®^ 488 (AF488, Thermo Fischer, Waltham, MA, USA) solution (50 μM dissolved in water) excited at the same laser power. For the focal volume calibration, a series of point FCS measurements was performed (ten measurements at different locations, each consisting of 15 repetitions of 10 s), and the data were fitted with a three-dimensional model including a triplet contribution. The structure parameter was typically around 6 to 9, and the diffusion time around 35 to 40 μs. The waist ω_0_ was calculated from the measured average diffusion time (τ_d,AF488_) and previously determined diffusion coefficient of the used dye at room temperature (D_AF488_ = 435 μm^2^s^−1^) [36], according to the following Equation (1):

(1) $$\omega_{0}=\sqrt{4\tau_{d,\mathrm{AF}488}D_{\mathrm{AF}488}}$$

Typical values were 200–250 nm. All measurements were performed at room temperature.

The sFCS analysis followed the procedure described previously [19,21,37]. Briefly, the TIFF files were imported and analyzed in Matlab using a custom-written code. All scanning lines were aligned as kymographs and divided in blocks of 1000 lines. In each block, lines were column-wise summed and the x position with maximum fluorescence was determined by fitting with a Gaussian function. This algorithm finds the position of the PM in each block and is used to align all lines to a common origin. The pixels corresponding to the membrane were defined as pixels which are within ±2.5 σ of the peak. In each line, these pixels were integrated, providing the membrane fluorescence time series F(t). A background correction was applied by subtracting the average pixel fluorescence value on the inner side of the membrane multiplied by 2.5 σ (in pixel units) from the membrane fluorescence, in blocks of 1000 lines [38]. In order to correct for depletion due to photobleaching, the fluorescence time series was fitted with a two-component exponential function and a mathematical correction was applied [17]. Finally, the normalized ACF was calculated according to Equations (2) and (3):

(2) $$G\left(\tau \right)=\frac{\langle \delta F\left(t\right)\delta F\left(t-\tau \right)\rangle }{\langle F{\left(t\right)\rangle }^{2}}$$

where

(3) δF=F(t)−〈F(t)〉

To avoid artefacts that can be caused by long-term instabilities of the system or single bright events, correlation functions were first calculated segment wise (10 segments per time trace), and segments with distortions were manually removed before averaging the correlation functions. Eventually, a model for two-dimensional diffusion in the membrane and a Gaussian focal volume geometry [37] was fitted to the ACF, as described in Equation (4):

(4) $$G\left(\tau \right)=\frac{1}{N}{\left(1+\frac{\tau }{\tau_{D}}\right)}^{-1/2}{\left(1+\frac{\tau }{\tau_{D}S^{2}}\right)}^{-1/2}$$

where τ_D_ denotes the diffusion time, and N the number of particles. The structure parameter S was fixed to the value of the daily based calibration measurement. Diffusion coefficients (D) were calculated using the calibrated waist $\omega_{0}$ of the focal volume: $D=\omega_{0}^{2}/4\tau_{D}$.

All resulting data were analyzed using GraphPad Prism 5.0 (GraphPad Software Inc., San Diego, CA, USA), and were displayed as box plots indicating the median values and with Tukey whiskers. Quantities in the main text are expressed as mean ± SD. Sample sizes and *p*-values are indicated in figure captions. Statistically significant differences between control and test samples were determined using one-way ANOVA analysis followed by the Bonferroni’s multiple comparisons test. A *p*-value < 0.01 was considered indicative of statistical significance.

## 3. Results and Discussion

### 3.1. EPC3 Increases Membrane Fluidity in Lipid Bilayer Models

In order to assess the influence of EPC3 on lipid–lipid interactions, first, we investigated its effects on controlled membrane models. In more detail, we prepared SLBs mimicking the general composition of the outer leaflet of the PM. These bilayers were composed of a natural mixture of PC (i.e., eggPC), sphingomyelin (bSM), and cholesterol (4/4/2 molar ratio). Such ternary lipids mixtures are known to separate into a liquid disordered (L_d_) and a liquid-ordered phase (L_o_), thus, providing a simple model for the study of phase separation occurring at the PM of living cells [22,39,40].

**Figure 1:**
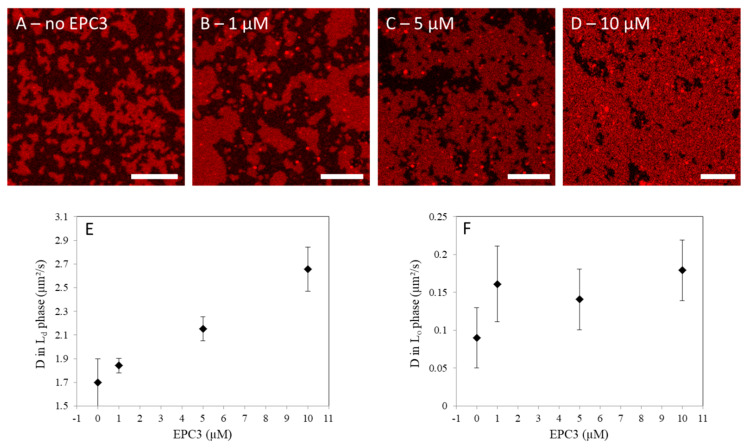
Effect of erufosine (EPC3) on phase-separating model membranes. (**A**–**D**) Representative confocal fluorescence microscopy images of eggPC/bSM/Chol 4/4/2 molar ratio supported lipid bilayers (SLBs) in the presence of 0, 1, 5, and 10 μM EPC3, respectively. The bilayers were labeled with 0.1 mol% Rhod-DOPE. Darker zones (devoid of Rhod-DOPE) indicate L_o_ regions of the SLB. Images were acquired at RT. Scale bars are 10 μm; (**E**–**F**) Values of the diffusion coefficients D measured via line-scan fluorescence correlation spectroscopy (lsFCS) in the L_d_, (**E**) and L_o_ phase (**F**) of eggPC/bSM/Chol 4/4/2 molar ratio SLBs in the presence of EPC3. The reported values refer to the diffusion of a fluorescent cholesterol analogue (TF-Chol, 0.01 mol%). Independent experiments were repeated in triplicate and averaged after normalization (see Materials and Methods). Error bars represent standard deviations.

In this experiment, first, we observed the effect of EPC3 on the general appearance of the L_o_/L_d_ phase separation in SLBs, via confocal fluorescence microscopy. For this purpose, SLBs were labeled with an unsaturated fluorescent lipid (Rhod-DOPE) which readily partitions in the L_d_ phase [41]. As apparent in Figure 1A, SLBs show bright patches enriched in Rhod-DOPE (i.e., L_d_ phase) and dark regions devoid of Rhod-DOPE (i.e., L_o_ phase). In the presence of increasing amounts of EPC3 in solution, the surface occupied by the L_d_ phase increases (Figure 1B–D, Figure S2). At the highest EPC3 concentration (i.e., 10 μM, Figure 1D), only small patches of L_o_ phase are still visible. These results indicate that EPC3 can effectively insert into the bilayer and destabilize the L_o_ phase. A similar induction of lipid mixing was also observed for other APLs in giant unilamellar vesicles [42].

Next, we characterized the degree of order of the lipid bilayer, in each phase. To this aim, we quantified the diffusion coefficient (D) of a fluorescent membrane probe (TF-Chol) both in the L_o_ and L_d_ phases. Lipid dynamics are, in general, connected with membrane order, with lower D values associated with tighter lipid packing and stronger lipid–lipid interactions [22]. Using lsFCS, we measured D for TF-Chol in the L_d_ (Figure 1E) and L_o_ phases (Figure 1F), in the presence of increasing concentrations of EPC3. In the absence of the drug, D was ca. 0.1 μm^2^/s in the ordered phase and ca. 20-fold higher in the disordered phase, as expected from previous experiments [39]. In the presence of EPC3, we observed a ca. 60% increase in lipid diffusion dynamics in the L_d_ phase. For the L_o_ phase, we observed a similar increase in D, although the data spread was larger in this case. On the one hand, these results clearly suggest that EPC3 has a fluidizing effect on the lipid bilayer that leads to a significant increase of the diffusion of membrane components. On the other hand, it is not possible to determine from these data whether EPC3 interacts preferentially with (and inserts in the bilayer through) the L_o_ or the L_d_ phase. Interestingly, saturated alkylphospholipids were suggested to partition at the boundary between L_o_ and L_d_ phases [42].

### 3.2. EPC3 Treatment Alters Phospholipid and Cholesterol Contents in Cell Membranes

To monitor the influence of EPC3 on the concentrations of the major phospholipid components and cholesterol in cell membranes (i.e., PM and internal membranes), we characterized the following two breast cancer cell models: the high-invasive MDA-MB-231 cell line and the low-invasive MCF-7 cell line. Both cell types were treated with EPC3 (IC_50_ and IC_75_) for 24 h and lipid amounts were measured by means of TLC and gas chromatography.

**Figure 2:**
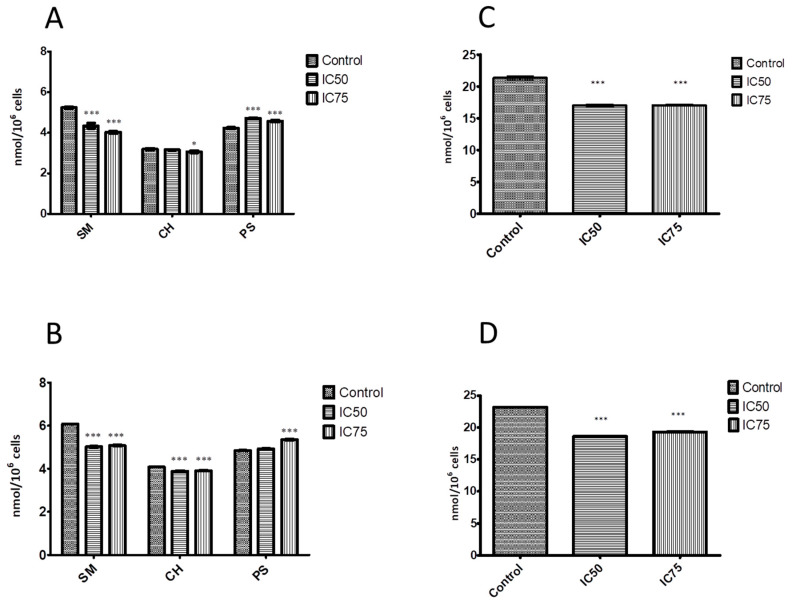
Effect of EPC3 on the level of sphingomyelin, cholesterol, phosphatidylserine, and phosphatidylcholine in cell membranes. (**A**) Amounts of sphingomyelin (SM), cholesterol (CH), and phosphatidylserine (PS) in MCF-7 cells treated with EPC3 for 24 h; (**B**) Amounts of sphingomyelin (SM), cholesterol (CH), phosphatidylserine (PS) in MDA-MB-231 cells treated with EPC3 for 24 h; (**C**) Amounts of phosphatidylcholine (PC) in MCF-7 cells treated with EPC3 for 24 h; (**D**) Amounts of phosphatidylcholine (PC) in MDA-MB-231 cells treated with EPC3 for 24 h. Data are obtained as means of three independent experiments. Error bars represent the standard deviations. Statistical significance is calculated against controls in each group using ANOVA one-way test and Bonferroni post-test. * *p* < 0.01 and *** *p* < 0.0001.

Some APLs are known to inhibit the synthesis of key phospholipids such as PC and SM [43,44,45,46]. Accordingly, we observed a similar effect in EPC3-treated MCF-7 and MDA-MB-231 cells (Figure 2A,B, respectively). The SM content significantly decreased with increasing concentration of EPC3 in both cell lines. For instance, the decrease of SM content after treatment with IC_75_ EPC3 was ~24% for MCF-7 cells and ~16% for MDA-MB-231 cells as compared with the control samples. This finding is in line with the observations of Marco and coworkers [47] who showed that treatment of human hepatoma HepG2 cell line with Miltefosine led to inhibition of SM metabolism. Disturbances in SM synthesis could cause the accumulation of ceramide and sphingosine, which regulate cellular functions such as proliferation, gene expression, differentiation, mitosis, cell survival, and apoptosis. For example, sphingosine and ceramide can induce apoptosis via the intrinsic apoptotic pathway by modulating the permeability of the mitochondrial membrane, thereby releasing proteins such as cytochrome C [48].

Furthermore, our results show an EPC3-induced reduction of PC amount. The effect was observed in both cancer cell lines (Figure 2C,D). In the MCF-7 cells treated with IC_75_ EPC3, the PC decrease was 20% as compared with the control samples. In the MDA-MB-231 cells, we observed a 17% decrease. Interestingly, one of the pathways for inducing apoptosis in cells treated with APLs is thought to be indeed the inhibition of PC synthesis. Our results support this hypothesis, as they show statistically significant reductions in PC levels at both EPC3 concentrations. Previous studies have demonstrated that Miltefosine also inhibited PC synthesis [44].

In contrast, the PS levels in the membranes of both treated cell lines appear to slightly increase (Figure 2A,B). Increased PS levels have also been previously observed in cancer cells as a response to chemotherapy or radiation treatment [49].

As shown in Figure 2A,B, treatment with EPC3 also resulted in a slight decrease in cholesterol levels. The membranes of the highly invasive cell line MDA-MB-231 were found to initially contain more cholesterol than those of the MCF-7 cells. These results are consistent with the hypothesis that high cholesterol levels in cell membranes can enhance cell migration in cancer models, including MDA-MB-231 cells [50,51,52]. Our data further show a reduction in cholesterol levels after treatment with EPC3 cells that is correlated with reduced cell survival, as we have previously observed [31]. Recent studies have indicated that the administration of membrane-active APLs such as edelfosine, erucylphosphocholine, and perifosine reduces the proliferation of HepG2 cells, disrupting cholesterol trafficking from the PM to intracellular membranes and decreasing the esterification of cholesterol [53]. Our data show that treatment of both MCF-7 and MDA-MB-231 cells with highly cytotoxic concentrations of EPC3 (IC_75_, [31]) resulted in a small but reproducible reduction in cholesterol levels (i.e., ~5%). The molar ratio of phospholipid-to-cholesterol content of low invasive untreated MCF-7 cells was ~9.7. After treatment with EPC3 (IC_75_), the ratio decreased to ~8.4 (unpaired t-test, *p* < 0.0004). The high-metastatic cell line MDA-MB-231 showed an average phospholipid-to-cholesterol ratio of ~8.4, which decreased to 7.6 upon treatment with IC_75_ EPC3 (unpaired t-test, *p* < 0.0002). Altered levels of (intra-)cellular lipids and cholesterol are linked to cancer aggressiveness [54,55,56]. More specifically, saturated lipids were suggested to reduce the fluidity and dynamics of the membrane and to increase resistance to conventional chemotherapy [57]. The presence and amounts of saturated sphingolipids (e.g., SM) are linked to the physical properties of ordered PM domains (e.g., raft domains) [58]. In addition, reducing cholesterol content with membrane-depleting agents or inhibitors of cholesterol synthesis (e.g., statins) was suggested to alter the structure of lipid rafts [57]. The consequent raft destabilization could, in turn, interfere with the proliferation and migration of tumor cells [59,60]. EPC3 treatment of both cancer cell lines induces a significant decrease in the amounts of specific cellular lipids, such as SM and cholesterol, as well as alterations in their relative molar ratios. Therefore, we argue that the cytotoxic effects of EPC3 could be linked to alterations in the lateral organization of cellular membranes and, in particular, to a decreased stability or amount of ordered lipid domains.

### 3.3. EPC3 Alters the Fluidity of the Plasma Membrane of Living Cells

In order to investigate the effects of EPC3 directly in the PM of living cells, we applied an approach based on the spectroscopic properties of two fluorescent molecules (i.e., Di-4-ANEPPDHQ and Laurdan) which are strongly sensitive to membrane order and lipid packing [25,26]. In the context of studies regarding the effect of APLs on lipid bilayers, Laurdan was used to detect and increase in the fluidity of model membranes induced by Miltefosine [61]. In this experiment, we directly characterized the PM of both MDA-MB-231 and MCF-7 breast cancer cell models. In previous studies, we observed that EPC3 caused increased cytotoxicity, apoptosis, and cytoskeleton reorganization especially in highly invasive MDA-MB-231 cells as compared with MCF-7 samples [62,63]. In addition, MDA-MB-231 cells showed cell cycle arrest after treatment with EPC3 [63].

The mechanism by which Laurdan and Di-4-ANEPPDHQ detect changes in the local membrane environment is similar for the two molecules, i.e., the less polar environment of the ordered bilayer phase (e.g., L_o_ phase or a raft-like domain in the PM) induces a blue shift in the emission maxima of both fluorescent probes. This shift can be quantified by calculating a ratiometric measurement of the fluorescence intensity observed in two spectral regions (or channels), known as a GP value. Higher GP values correspond to a relatively higher fluorescence emission in the shorter-wavelength spectral region, and therefore a higher degree of membrane order and tighter lipid packing [26].

The MDA-MB-231 and MCF-7 cells were treated with the corresponding IC_50_ and IC_75_ EPC3 concentrations and were observed via confocal fluorescence microscopy after 24, 48, and 72 h. The EPC3 treatment induced, in general, decreased cell viability, accompanied by morphological changes which, eventually, led to apoptosis [31]. Next, we focused our analysis on early apoptotic cells, i.e., cells which do not yet appear morphologically significantly different from those in the control samples. For such cells, it was possible to clearly identify the PM and perform a GP analysis of the fluorescence signal (Figure S1).

**Figure 3:**
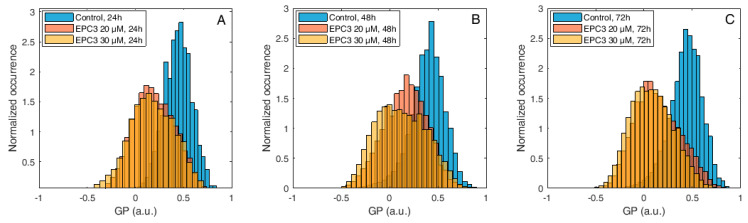
Di-4-ANEPPDQH generalized polarization (GP) values measured at the plasma membrane (PM) of MDA-MB-231 cells after EPC3 treatment. Normalized histograms of GP values measured in pixels belonging to the PM of MDA-MB-231 cells labeled with Di-4-ANEPPDQH. Cells were treated with 20 μM (orange bars) or 30 μM (yellow bars) EPC3. GP values measured in cells not treated with EPC3 are shown as blue bars. Fluorescence intensity values were acquired 24 h (**A**), 48 h (**B**), and 72 h (**C**) after the addition of EPC3. For each condition, GP values were pooled from ca. 50 ROIs selected at the PM of distinct cells, in two independent experiments. The total number of calculated GP values (and measured pixels) for each experimental condition was between ca. 10,000 and 40,000. Measurements were performed at RT. Representative images of treated cells and ROIs are shown in Figures S1 and S3.

Figure 3 shows normalized histograms for Di-4-ANEPPDQH GP values measured in MDA-MB-231 cells, in the presence of 20 μM or 30 μM EPC3. In all cases, the PM region of several cells was manually selected and the GP values for each pixel within these ROIs were calculated, as described in the Materials and Methods section and Figure S3A–D.

**Figure 4:**
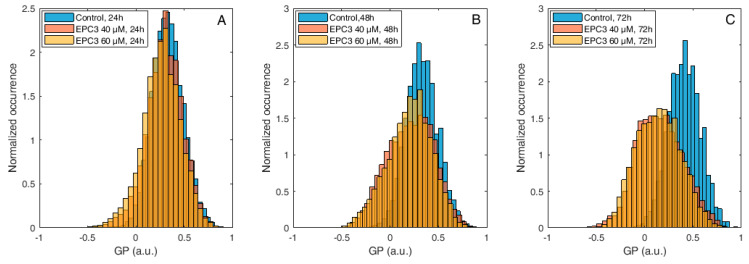
Di-4-ANEPPDQH GP values measured at the PM of MCF-7 cells after EPC3 treatment. Normalized histograms of GP values measured in pixels belonging to the PM of MCF-7 cells labeled with Di-4-ANEPPDQH. Cells were treated with 40 μM (orange bars) or 60 μM (yellow bars) EPC3. GP values measured in cells not treated with EPC3 are shown as blue bars. Fluorescence intensity values were acquired 24 h (**A**), 48 h (**B**), and 72 h (**C**) after the addition of EPC3. For each condition, GP values were pooled from ca. 50 ROIs selected at the PM of distinct cells, in two independent experiments. The total number of calculated GP values (and measured pixels) for each experimental condition was between ca. 10,000 and 30,000. Measurements were performed at RT. Representative images of treated cells and ROIs are shown in Figures S1 and S4.

As shown in Figure 3, Figure S3, and Table S1, the fluorescence emission of Di-4-ANEPPDQH indicates that EPC3 treatment of cancerous cells induces a time-dependent increase of PM disorder. Only minor differences were observed between IC_50_ and IC_75_ EPC3 concentrations (i.e., 20 and 30 μM for MDA-MB-231 cells, respectively). Performing the same experiment using MCF-7 cells (Figure 4, Figure S4, and Table S2), we observed that EPC3-induced membrane order alterations are stronger and occur faster in MDA-MB-231 cells.

In the case of MCF-7 cells, in fact, no significant alteration in membrane order was observed after 24 h of treatment, both at IC_50_ and IC_75_ EPC3 concentrations (i.e., 40 and 60 μM for MCF-7 cells, respectively). After 48 and 72 h, however, the GP distribution appeared significantly shifted to lower values, independently from the EPC3 concentration (Figure 4B–C, Figure S4, and Table S2).

Next, we employed a complementary assay quantifying the GP parameter, following analogous treatment of MDA-MB-231 and MCF-7 cells, using Laurdan as the fluorescent probe instead of Di-4-ANEPPDHQ (Figures S1I,L and S5).

**Figure 5:**
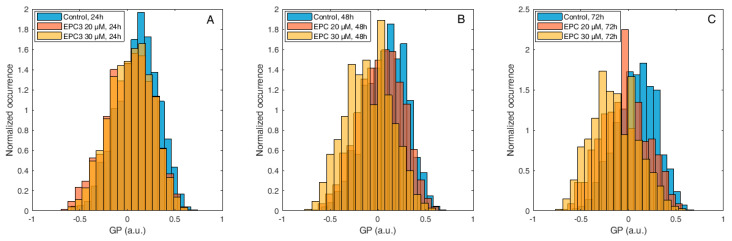
Laurdan GP values measured at the PM of MDA-MB-231 cells after EPC3 treatment. Normalized histograms of GP values measured in pixels belonging to the PM of MDA-MB-231 cells labeled with Laurdan. Cells were treated with 20 μM (orange bars) or 30 μM (yellow bars) EPC3. GP values measured in cells not treated with EPC3 are shown as blue bars. Fluorescence intensity values were acquired 24 h (**A**), 48 h (**B**), and 72 h (**C**) after the addition of EPC3. For each condition, GP values were pooled from ca. 50 ROIs selected at the PM of distinct cells, in two independent experiments. The total number of calculated GP values (and measured pixels) for each experimental condition was between ca. 10,000 and 40,000. Measurements were performed at RT. Representative images of treated cells and ROIs are shown in Figures S1 and S5.

The results shown in Figure 5, Figure S5, and Table S3 indicate the presence of a concentration-dependent decrease in membrane order after 48 and 72 h treatment with EPC3. We did not observe prominent alterations of the cell membrane order after 24 h treatment. These observations are qualitatively similar to those performed using Di-4-ANEPPDQH as the fluorescent probe. In addition, in analogy to the results shown in Figure 4 (i.e., labeling of MCF-7 cells with Di-4-ANEPPDQH), the observed changes in lipid packing were larger in MDA-MB-231 than in MCF-7 cells (Figures S6 and S7, and Table S4).

In summary, fluorescence microscopy measurements based on both Laurdan and Di-4-ANEPPDQH labeling indicate a decrease in PM lipid packing and order induced by EPC3, especially in the case of MDA-MB-231 cells. It is worth noting that, since the GP analysis was performed on early apoptotic cells (i.e., morphologically still comparable to untreated cells), the actual decrease in average membrane order for the whole cell population could be even more significant than what was suggested by our results. Additionally, the observed effects were much more noticeable when Di-4-ANEPPDHQ was used, instead of Laurdan. This difference could stem from two different factors. First, on the one hand, as previously described [64], Di-4-ANEPPDQH is more effective in specifically labeling the PM. Laurdan, on the other hand, penetrates more easily into the cytosol and labels also intracellular structures (see Figure S1), thus making ROI selections more difficult and less precise, while generally increasing the spread in observed GP values. Second, Di-4-ANEPPDQH and Laurdan are sensitive to different properties of the lipid bilayer [25]. While Laurdan is sensitive to lipid packing, the spectroscopic properties of Di-4-ANEPPDQH are influenced by other factors, such as cholesterol content of the membrane or internal electric dipole potential of the bilayer. Therefore, it is tempting to assume that the changes in membrane order (observed by using Di-4-ANEPPDQH rather than Laurdan) could be connected to specific alterations in membrane compositions brought about by EPC3. This hypothesis is in agreement with our observation that EPC3 alters the concentration of key lipid components of the PM (e.g., cholesterol and SM) which play an important role in membrane lateral organization (Figure 2). Similarly, it has been previously shown that other APLs could influence lipid metabolism and lipid-mediated signaling cascades. However, our experiments on model membranes clearly indicate that EPC3 can also directly influence lipid–lipid interactions and membrane fluidity, even in the absence of any alteration in membrane composition (Figure 1). Therefore, we conclude that EPC3 modulates the physical properties of the PM, for example, by directly influencing the interactions between cholesterol and other lipids or altering the internal electric dipole potential of the bilayer. Additional effects due to alterations in lipid metabolism and PM composition could also be involved in vivo.

### 3.4. EPC3 Increases Diffusion Dynamics of Trans-Membrane Proteins in the PM

We next investigated whether the alterations in lipid–lipid interactions, as well as lipid composition caused by EPC3 treatment discussed in the previous paragraphs can have a specific influence on the behavior of membrane proteins. Therefore, we investigated the diffusive dynamics of two illustrative transmembrane proteins, namely the human EGF receptor (hEGFR) and the hemagglutinin receptor from influenza A FP virus (FPV-HA), in MDA-MB-231 cells treated with 30 μM EPC3 (IC_75_). The hEGFR is a well-characterized membrane protein which partitions in lipid domains of the PM [65]. The transmembrane glycoprotein HA of the influenza A virus is known to form trimers at the PM, where it localizes in raft domains [66,67].

**Figure 6:**
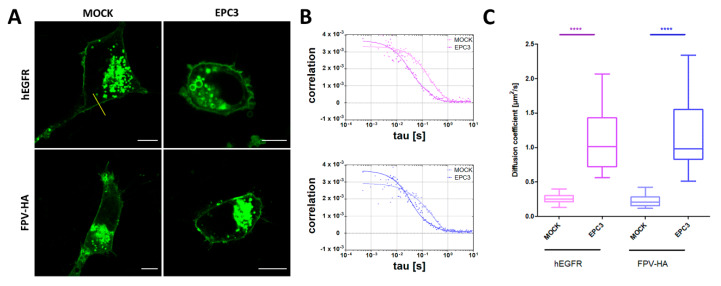
Quantification of protein diffusion in EPC3-treated cells via scanning fluorescence correlation spectroscopy (sFCS). (**A**) Representative confocal fluorescence microscopy images of MDA-MB-231 cells expressing hEGFR-EGFP (upper row), and FPV-HA-EGFP (lower row), in the absence of EPC3 or after 48 h incubation with EPC3. The yellow line in (**A**) represents an example of scanning path used for sFCS measurements. Scale bars are 10 μm; (**B**) Representative sFCS autocorrelation functions and fit curves obtained for cells expressing hEGFR-EGFP (upper graph) and FPV-HA-mEGFP (lower graph), in the absence of EPC3, or after 48 h incubation with EPC3. Fit curves (solid line) were obtained by fitting a two-dimensional diffusion model to the data, as described in the Methods section; (**C**) Shows box plots of diffusion coefficients calculated from sFCS diffusion times, pooled from three independent experiments (each consisting of measurements on 20 cells). The value of diffusion coefficient D for hEGFR-EGFP significantly increased from 0.26 ± 0.08 to 1.1 ± 0.5 μm^2^s^−1^ after EPC3 treatment. The value of diffusion coefficient D for FPV-HA-mEGFP significantly increased from 0.22 ± 0.08 to 1.2 ± 0.5 μm^2^s^−1^ after EPC3 treatment. All measurements were performed at RT. **** *p* < 0.0001.

As shown in the previous paragraph, EPC3 (in this case, 30 μM in MDA-MB-231 cells) strongly influences the physical state of the PM, as reported by Di-4-ANEPPDQH. The two examined proteins were labeled with a fluorescent protein (GFP) and visualized via confocal fluorescence microscopy. As shown in Figure 6A, EPC3 does not significantly alter the localization of either protein at the PM. Then, we applied sFCS to quantify the diffusion dynamics of the two fluorescently labeled proteins. Figure 6B shows representative autocorrelation curves for the fluorescence signal recorded for hEGFR (upper panel) and FPV-HA (lower panel), also in the presence of EPC3.

Both membrane proteins diffuse in the PM of untreated cells with a diffusion coefficient D ~0.2 μm^2^/s, as expected from previous experiments [19,20,21]. In the presence of EPC3, we observed an average ~5-fold increase in diffusion dynamics, both for hEGFR and FPV-HA (Figure 6C). This appears to be due to a direct EPC3-lipid bilayer interaction (rather than, e.g., cytoskeleton reorganization), as suggested by measurements in PM-derived giant lipid vesicles (Figure S8 and Table S5). In fact, a moderate increase in FPV-HA-EGFP dynamics can be observed already 1 h after EPC3 treatment. Then, the diffusion coefficient D increases further during the course of 48 h treatment, both for proteins at the PM and in GPMVs (Figure S8 and Table S5). Changes in protein diffusion were previously observed as a consequence of alterations in lipid–lipid and lipid–protein interactions. For example, it was shown that hEGFR and HA diffusion coefficients increase as a result of alterations in membrane composition or protein dissociation from membrane domains [28,65,68]. Our data suggest that EPC3 could alter the physical properties of the PM through two different processes. Initially, EPC3 modifies lipid–lipid interactions, as seen in model membranes (Part 3.1) and in cell models 1 h after EPC3 treatment. Later on, alterations in lipid metabolism and membrane composition also induced by EPC3 (Part 3.2) could further increase membrane fluidity and dynamics, acting in synergy with the initial effect.

## 4. Conclusions

The increased amount of saturated lipids and ordered lipid domains in tumor cells (as compared with healthy cells) [69] correlates with a reduction in membrane fluidity/dynamics and an increase in chemotherapy resistance [57,70]. In cancer cells, a wide range of signaling proteins and receptors regulating pro-oncogenic and apoptotic pathways are localized in ordered lipid domains (e.g., raft domains) [6]. It is now accepted that APLs exert their effect by interacting with such domains, due to their participation in the regulation of cell survival and cell death pathways [71]. In this context, we show, for the first time, that EPC3 causes alterations in cellular lipid composition and a significant increase in PM disorder through, at least, two separate mechanisms. These effects appear to be stronger in the high-invasive cancer cell line MDA-MB 231 and to have a direct effect on the dynamics of membrane components, as demonstrated by our measurements of membrane protein diffusion. Increased protein diffusion dynamics likely affects the likelihood of protein–protein interactions, and therefore could modulate signaling pathways involved in cell survival. Furthermore, it is possible that the decrease in lipid packing corresponds to a higher permeability of the PM, and thus an increased intake of EPC3 or other drugs through the cell membrane.

## Supplementary Materials

The following are available online at https://www.mdpi.com/2218-273X/10/5/802/s1, Figure S1: Typical fluorescence microscopy images of cells samples used in this work, Figure S2: Variation of ordered phase surface extension in response to EPC3 treatment, Figure S3: Analysis of Di-4-ANEPPDHQ GP values measured at the PM of MDA-MB-231, Figure S4: Analysis of Di-4-ANEPPDHQ GP values measured at the PM of MCF-7, Figure S5: Analysis of Laurdan GP values measured at the PM of MDA-MB-231, Figure S6: Laurdan GP values measured at the PM of MCF-7 cells after EPC3 treatment, Figure S7: Analysis of Laurdan GP values measured at the PM of MCF-7 cells, Figure S8: sFCS measurements in giant plasma membrane vesicles (GPMV), Table S1: Di-4-ANEPPDHQ GP values (mean and standard deviation) measured at the PM of MDA-MB-231, Table S2: Di-4-ANEPPDHQ GP values (mean and standard deviation) measured at the PM of MCF-7, Table S3: Laurdan GP values (mean and standard deviation) measured at the PM of MDA-MB-231, Table S4: Laurdan GP values (mean and standard deviation) measured at the PM of MCF-7 cells after EPC3 treatment, Table S5: Diffusion coefficients of FPV-HA-EGFP measured via sFCS, at different time points after treatment with EPC3.
