# Supplementary material for: Effect of Erufosine on Membrane Lipid Order in Breast Cancer Cell Models
Source: Biomolecules. 2020 May 22;10(5):802. doi: 10.3390/biom10050802 (PMC7277205; doi:10.3390/biom10050802)
Supplement: Supplementary file 1 [file biomolecules-10-00802-s001.pdf]

SUPPLEMENTARY INFORMATION

Tzoneva R., Stoyanova T. et al.

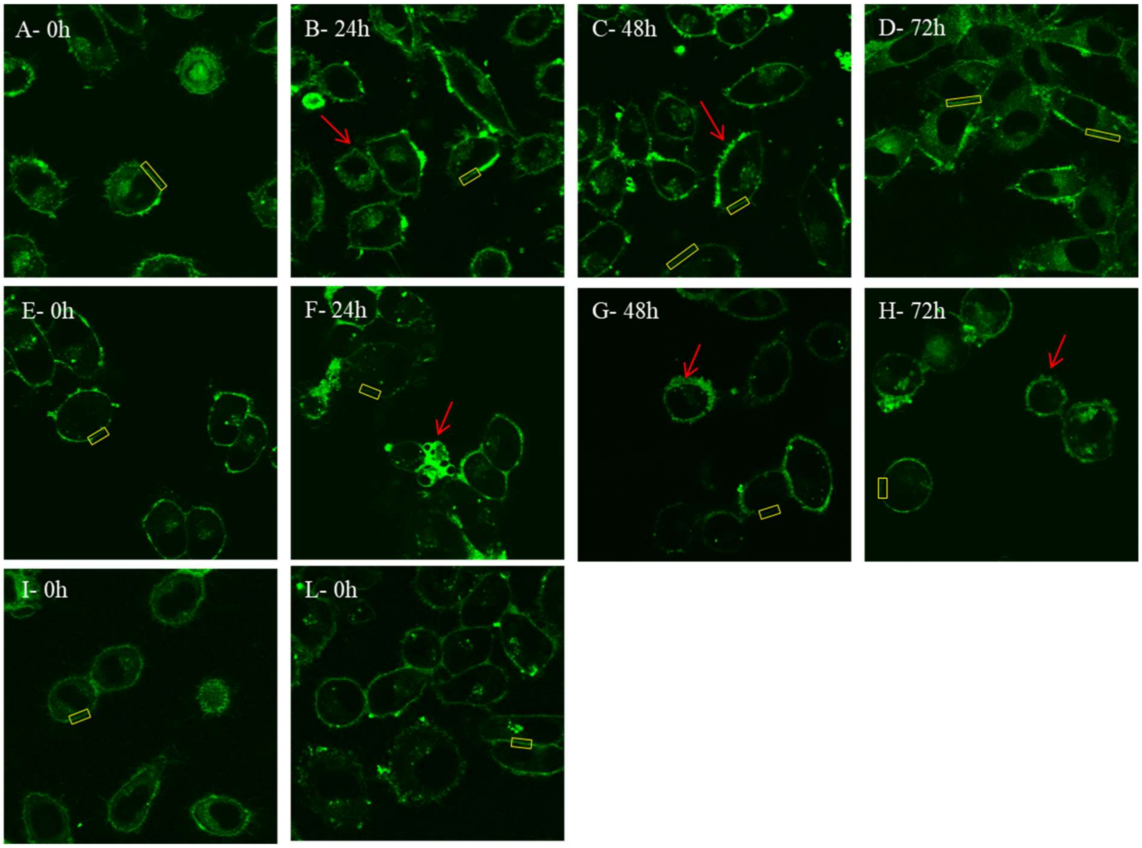

Figure S1. Typical fluorescence microscopy images of cells samples used in this work. Confocal microscopy images of MDA-MB 231 (**A-D**, **I**) and MCF-7 cells (**E-H**, **L**). Cells were labelled with Di-4-ANEPPDHQ (**A-H**) or with Laurdan (**I** and **L**). Samples are shown before treatment (**A**, **E**, **I** and **L**) and after treatment with IC_{75} EPC3 (24h: **B** and **F**; 48h: **C** and **G**; 72h: **D** and **H**). The yellow rectangles represent examples of ROIs selected for general polarization analysis. Red arrows indicate examples of morphological alterations in the plasma membrane of treated cells. These regions were in general not included in further analysis. All images are 106 μm x 106 μm.

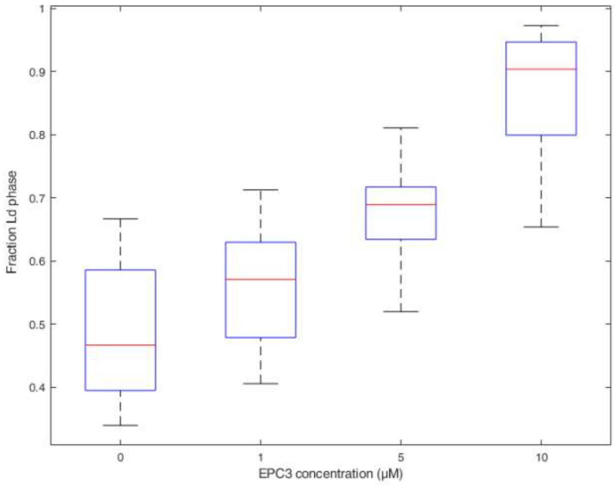

Figure S2. Variation of ordered phase surface extension in response to EPC3 treatment. Confocal fluorescence microscopy images of EggPC/bSM/Chol 4/4/2 molar ratio SLBs were acquired in the presence of 0, 1, 5 and 10 μM EPC3 (see Figure 1 A-D of the main text). Rhod-DOPE (0.1 mol%) was used to label the L_d phase. ROIs were selected to exclude e.g. bilayer defects and were filtered using a median filter. Subsequently, ROIs were binarized using a threshold that ensured a net separation into two phases and minimal pixel noise. The ratio between the number of pixels containing high intensity (corresponding to the L_d phase) and the total amount of pixels in the ROI is represented as box plot. A total amount of 16-26 images were analyzed for each EPC3 concentration.

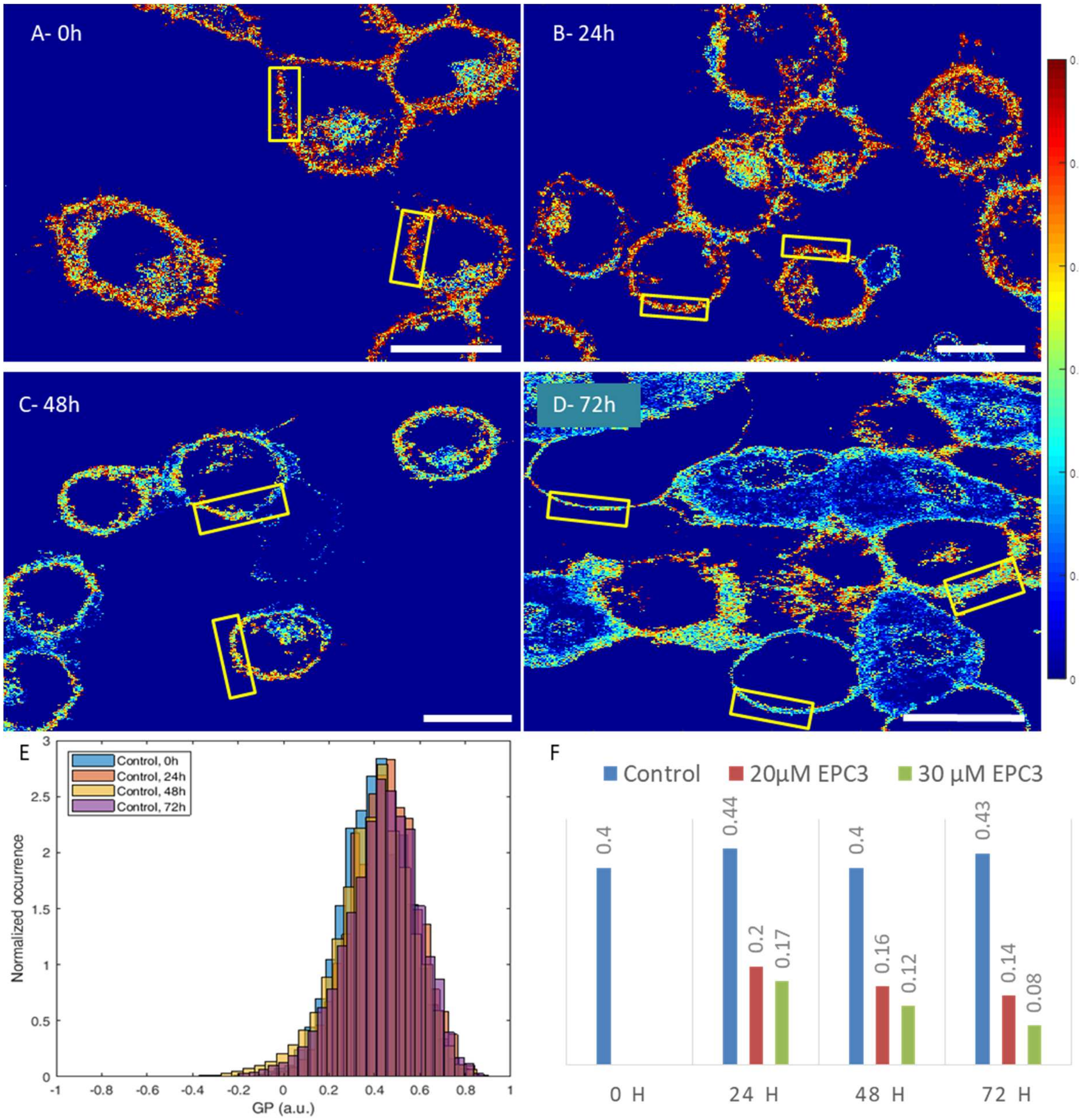

Figure S3. Analysis of Di-4-ANEPPDHQ GP values measured at the PM of MDA-MB-231. (**A**-**D**) Representative GP images of MDA-MB-231 cells labeled with Di-4-ANEPPDHQ and treated with IC_{75} EPC3, after 0, 24, 48 and 72h. Yellow rectangles represent examples of ROIs selected for GP quantification. (**E**) GP values for all the control samples at different time points (see Fig. 3 in the main text), including the data acquired before the beginning of the EPC3 treatment (Control, 0h). (**F**) Average values of the normalized histograms shown in Fig. 3 and in Table S1, as a function of treatment time.

Table S1. Di-4-ANEPPDHQ GP values (mean and standard deviation) measured at the PM of MDA-MB-231.

	Mean	Std. dev.
Control 0h	**0.40**	**0.15**
Control 24h	**0.44**	**0.15**
IC50 24h	**0.20**	**0.22**
IC75 24h	**0.17**	**0.24**
Control 48h	**0.40**	**0.17**
IC50 48h	**0.16**	**0.22**
IC75 48h	**0.12**	**0.26**
Control 72h	**0.43**	**0.17**
IC50 72h	**0.14**	**0.23**
IC75 72h	**0.08**	**0.22**

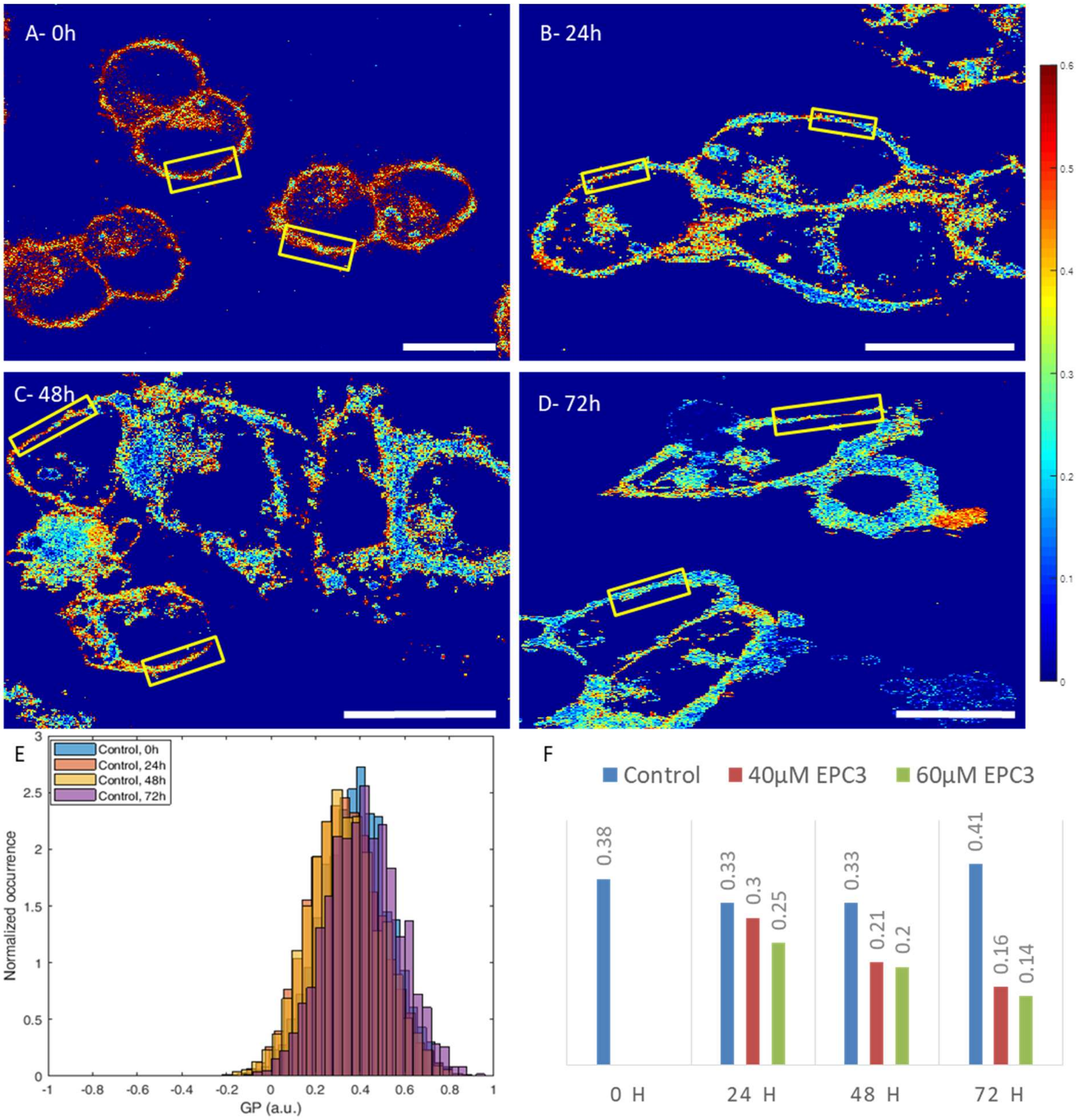

Figure S4. Analysis of Di-4-ANEPPDHQ GP values measured at the PM of MCF-7. (**A**-**D**) Representative GP images of MCF-7 cells labeled with Di-4-ANEPPDHQ and treated with IC_{75} EPC3, after 0, 24, 48 and 72h. Yellow rectangles represent examples of ROIs selected for GP quantification. (**E**) GP values for all the control samples at different time points (see Fig. 4 in the main text), including the data acquired before the beginning of the EPC3 treatment (Control, 0h). (**F**) Average values of the normalized histograms shown in Fig. 4 and in Table S2, as a function of treatment time.

Table S2. Di-4-ANEPPDHQ GP values (mean and standard deviation) measured at the PM of MCF-7.

	Mean	Std. dev.
Control 0h	**0.38**	**0.16**
Control 24h	**0.33**	**0.16**
IC50 24h	**0.30**	**0.19**
IC75 24h	**0.25**	**0.20**
Control 48h	**0.33**	**0.16**
IC50 48h	**0.21**	**0.24**
IC75 48h	**0.20**	**0.23**
Control 72h	**0.41**	**0.16**
IC50 72h	**0.16**	**0.24**
IC75 72h	**0.14**	**0.23**

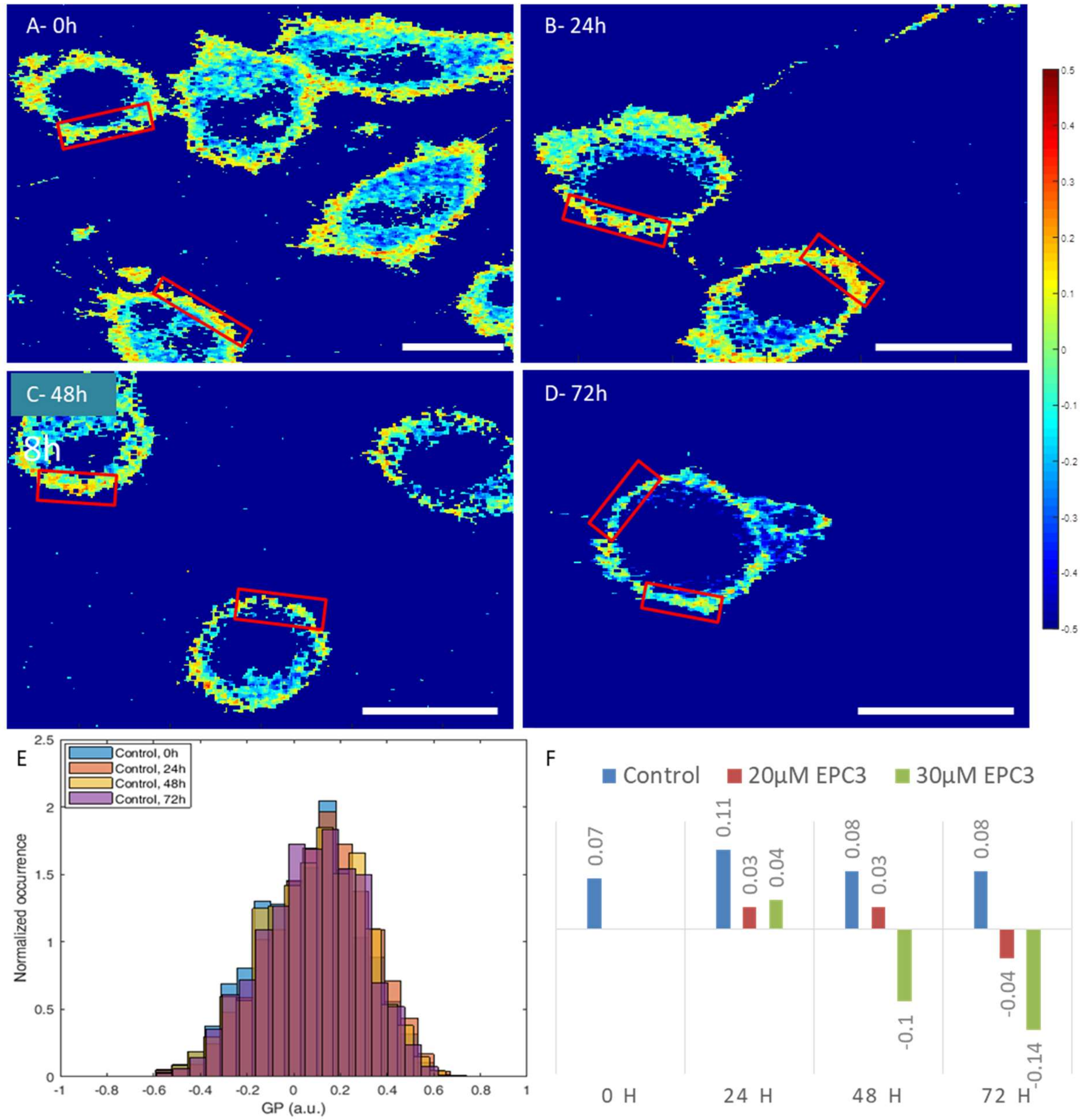

Figure S5. Analysis of Laurdan GP values measured at the PM of MDA-MB-231. (**A**-**D**) Representative GP images of MDA-MB-231 cells labeled with Laurdan and treated with IC_{75} EPC3, after 0, 24, 48 and 72h. Red rectangles represent examples of ROIs selected for GP quantification. (**E**) GP values for all the control samples at different time points (see Fig. 5 in the main text), including the data acquired before the beginning of the EPC3 treatment (Control, 0h). (**F**) Average values of the normalized histograms shown in Fig. 5 and in Table S3, as a function of treatment time.

Table S3. Laurdan GP values (mean and standard deviation) measured at the PM of MDA-MB-231.

	Mean	Std. dev.
Control 0h	**0.07**	**0.22**
Control 24h	**0.11**	**0.22**
IC50 24h	**0.03**	**0.24**
IC75 24h	**0.04**	**0.23**
Control 48h	**0.08**	**0.22**
IC50 48h	**0.03**	**0.23**
IC75 48h	**-0.10**	**0.24**
Control 72h	**0.08**	**0.21**
IC50 72h	**-0.04**	**0.24**
IC75 72h	**-0.14**	**0.24**

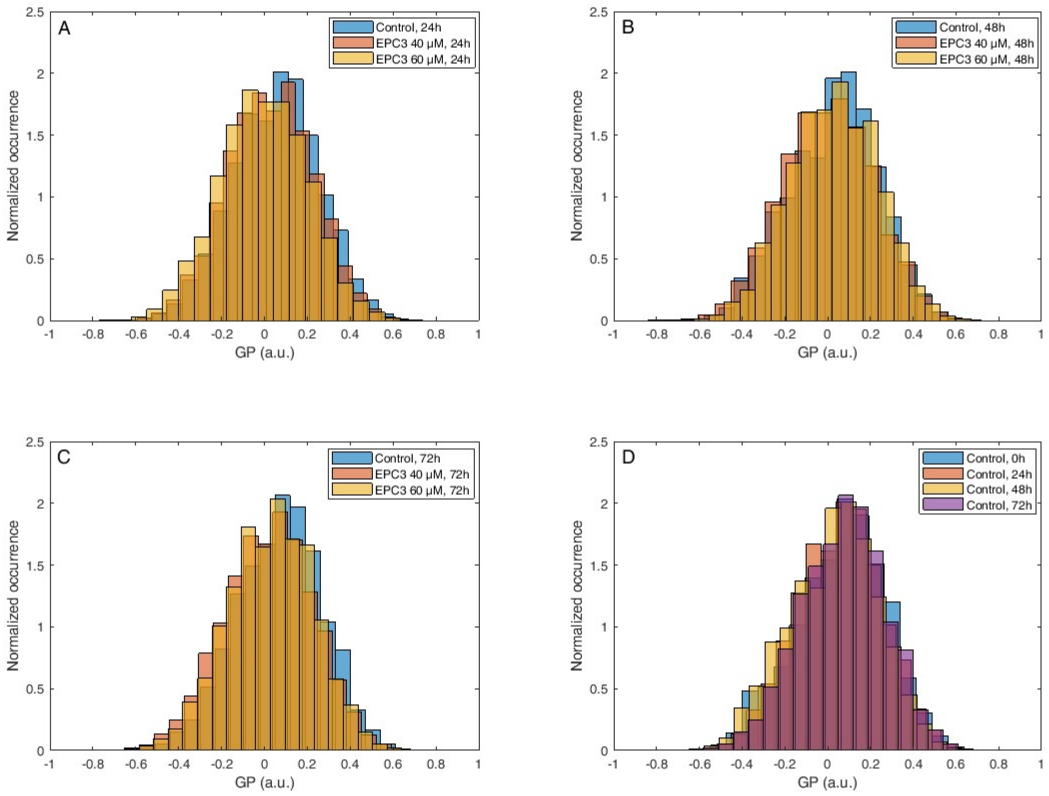

Figure S6. Laurdan GP values measured at the PM of MCF-7 cells after EPC3 treatment. (**A-C**) normalized histograms of GP values measured in pixels belonging to the PM of MCF-7 cells labelled with Laurdan. Cells were treated with 40 μM (orange bars) or 60 μM (yellow bars) EPC3. GP values measured in cells not treated with EPC3 are shown as blue bars. Fluorescence intensity values were acquired 24 h (**A**), 48 h (**B**) and 72 h (**C**) after the addition of EPC3. (**D**) GP values for all the control samples at different time points, including the data acquired before the beginning of the EPC3 treatment (Control, 0h). For each condition, GP values were pooled from ca. 50 ROIs selected at the PM of distinct cells, in two independent experiments. The total number of calculated GP values (and measured pixels) for each experimental condition was between ca. 10000 and 30000. Measurements were performed at RT.

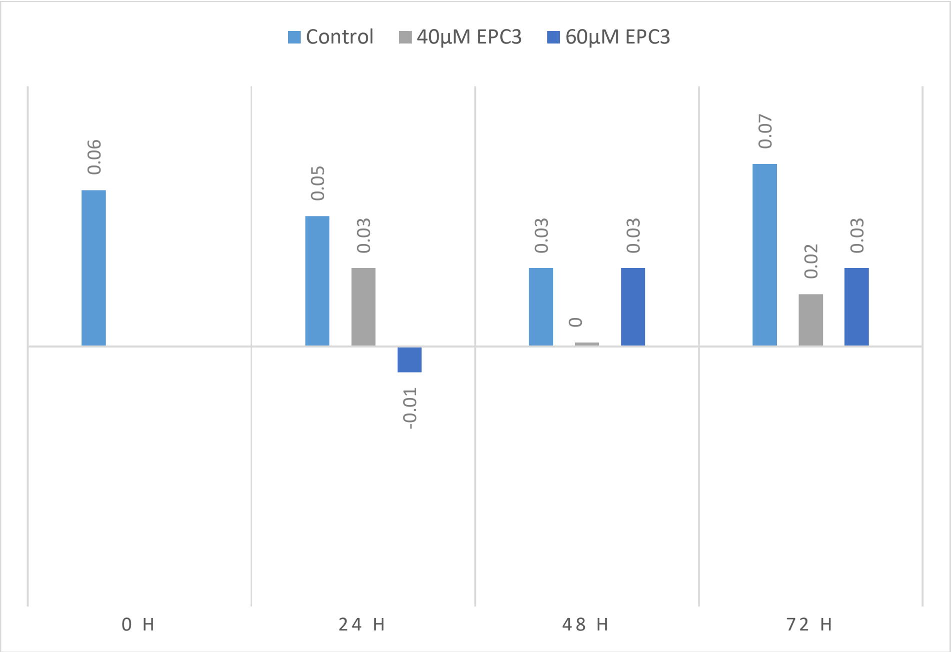

Figure S7. Analysis of Laurdan GP values measured at the PM of MCF-7 cells. Average values of the normalized histograms shown in Fig. S6 and in Table S4, as a function of treatment time.

Table S4. Laurdan GP values (mean and standard deviation) measured at the PM of MCF-7 cells after EPC3 treatment.

	Mean	SD
Control 0h	**0.06**	**0.21**
Control 24h	**0.05**	**0.20**
IC50 24h	**0.03**	**0.20**
IC75 24h	**-0.01**	**0.21**
Control 48h	**0.03**	**0.21**
IC50 48h	**0.00**	**0.21**
IC75 48h	**0.03**	**0.20**
Control 72h	**0.07**	**0.20**
IC50 72h	**0.02**	**0.21**
IC75 72h	**0.03**	**0.20**

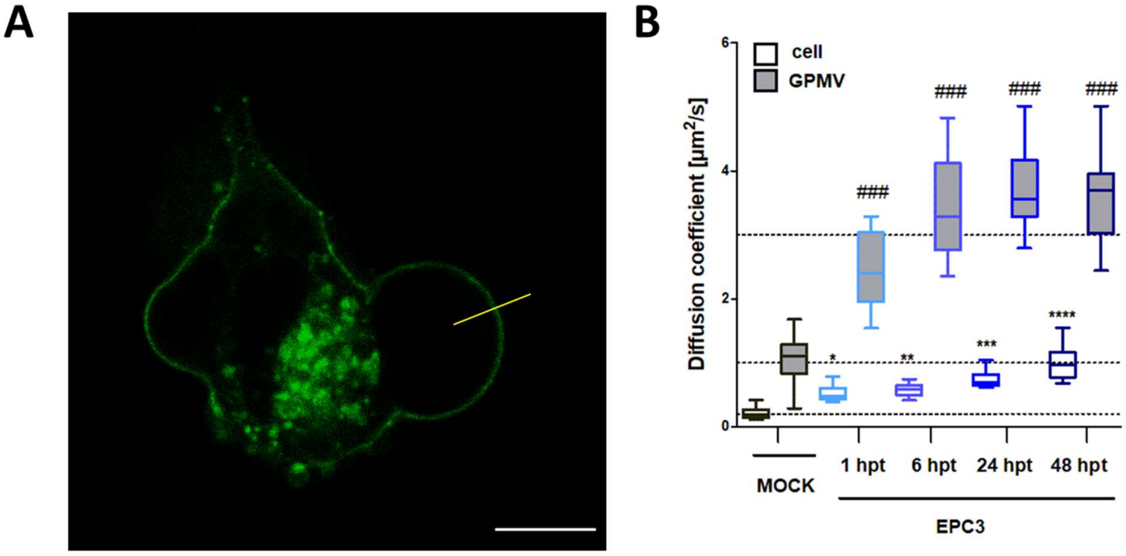

Figure S8. sFCS measurements in giant plasma membrane vesicles (GPMV). MDA-MB-231 cells were seeded on 35-mm dishes (CellVis, Mountain View, CA) with optical glass bottom. After 24 h, cells were transfected as described in Par. 2.9 of the main text. After 16 h, cells were treated with medium containing 30 μM EPC3 (IC_{75}) for 1 h, 6 h, and 24 h. After EPC3-treatment, GPMVs were produced following the *N*-ethylmaleimide (NEM) protocol (1). Briefly, cells were washed twice with GPMV buffer (10 mM HEPES, 150 mM NaCl, 2 mM CaCl2, pH 7.4) and then incubated in NEM-GPMV-Buffer (2 mM NEM, 10 mM HEPES, 150 mM NaCl, 2 mM CaCl2, pH 7.4) for 1 h at 37°C. Afterwards, sFCS measurements at the cell PM or in GPMVs were performed as described in Par. 2.9. To minimize out-of-focus movement or drift, GPMVs still attached to cells were selected for analysis.

(A) Representative image of GPMVs originating from a MDA-MB-231 cell expressing FPV-HA-EGFP. The yellow line indicates a typical scanning path used for sFCS measurements. Scale bar is 10 μm. (**B**) Box plots of diffusion coefficients D of FPV-HA-EGFP, after different amounts of hours post EPC3 treatment (hpt). The value of D significantly increases in the PM (from 0.21 ± 0.08 $\mu m^2/s$ to 1.0 ± 0.3 $\mu m^2/s$), and in GPMVs (from 1.1 ± 0.3 $\mu m^2/s$ to 3.7 ± 0.6 $\mu m^2/s$). Each group includes measurements of 20 to 26 cells or GPMVs. All measurements were performed at RT. Statistical tests were performed using one-way ANOVA followed by post-hoc Student's t-test with Bonferroni multiple comparison test. *$p < 0.01$ vs MOCK - cell, **$p < 0.005$ vs MOCK - cell, ***$p < 0.001$ vs MOCK - cell, #$p < 0.01$ vs MOCK - GPMV, ##$p < 0.005$ vs MOCK - GPMV, ###$p < 0.001$ vs MOCK - GPMV

Table S5. Diffusion coefficients of FPV-HA-EGFP measured via sFCS, at different time points after treatment with EPC3 (see Fig. S8).

	Number of examined cells/GPMVs	Diffusion coefficient (mean ± SD) [$\mu m^2/s$]	
		Cell PM	GPMV
MOCK	**20/26**	**0.21 ± 0.08**	**1.1 ± 0.3**
EPC3 1 hpt	**26/24**	**0.52 ± 0.11**	**2.5 ± 0.6**
EPC3 6 hpt	**20/20**	**0.65 ± 0.10**	**3.5 ± 0.8**
EPC3 24 hpt	**20/24**	**0.75 ± 0.12**	**3.7 ± 0.6**
EPC3 48 hpt	**20/24**	**1.0 ± 0.3**	**3.6 ± 0.7**

1. **Sezgin E, Kaiser HJ, Baumgart T, Schwille P, Simons K, Levental I.** 2012. Elucidating membrane structure and protein behavior using giant plasma membrane vesicles. Nat Protoc **7:**1042-1051.
